# Acupuncture therapy for Parkinson’s disease: a case report demonstrating symptomatic improvement without medication

**DOI:** 10.3389/fneur.2023.1330054

**Published:** 2024-01-29

**Authors:** Suying Lei, Qing Liu, IanI Leong, Jingqi Fan, YauKeung Tsang, Xin Liu, Xiaoyan Xu, Lixing Zhuang

**Affiliations:** ^1^The First Clinical School of Medicine, Guangzhou University of Chinese Medicine, Guangzhou, China; ^2^School of Acupuncture and Rehabilitation, Guangzhou University of Chinese Medicine, Guangzhou, China; ^3^The First Affiliated Hospital of Guangzhou University of Chinese Medicine, Guangzhou, China

**Keywords:** acupuncture therapy, Parkinson’s disease, alternative treatment, case report, symptomatic improvement

## Abstract

**Background:**

Parkinson’s disease (PD) often necessitates immediate medical intervention following diagnosis. In recent years, there has been a noticeable increase in clinical investigations assessing the efficacy of acupuncture in PD, with many studies reporting positive outcomes. Ethical guidelines commonly endorse pharmaceutical therapies for PD, leading ongoing research to combine acupuncture with standard drug-based treatments. At present, there is a conspicuous absence of dedicated clinical research exclusively examining the independent impact of acupuncture on PD treatment.

**Case:**

In a clinical observation, we documented a case involving a 75-year-old male displaying progressive, characteristic PD symptoms, including evident limb tremors, rigidity, bradykinesia, fatigue, and additional non-motor symptoms. The patient received a confirmed diagnosis of PD. Due to the refusal of the patient to take medication, we exclusively administered acupuncture therapy. The outcomes indicated a noteworthy enhancement in the clinical symptoms of the patient solely through acupuncture intervention.

**Conclusion:**

This case affirms that using acupuncture in isolation significantly improved both the motor and non-motor symptoms in the patient. Acupuncture could potentially serve as an alternative therapy for patients who decline or are intolerant to anti-Parkinson drugs. However, further studies are needed to assess its long-term efficacy. This case report obtained approval from the Ethics Committee of the First Affiliated Hospital of Guangzhou University of Chinese Medicine (Ethics number: K-2023-127).

## 1 Introduction

Parkinson’s disease (PD) stands as a prevalent neurodegenerative disorder (1) with treatment challenges that have yet to demonstrate the capability to halt long-term disease progression (2). Currently, pharmaceutical therapy remains the primary approach for managing PD (3, 4).

Traditional Chinese medicine (TCM) originally outlined symptoms akin to PD as shaking palsy in Huangdi Neijing around 100 A.D., maintaining a historical presence in the care of PD patients for thousands of years. Acupuncture, an ancient technique dating back 2000 years within TCM (5), has gained widespread utilization by physicians and patients globally in alleviating clinical symptoms in PD. Recent years have witnessed a discernible rise in clinical investigations exploring the use of acupuncture for PD, often reporting positive outcomes (6).

Furthermore, animal studies have provided a scientific basis, supporting the potential of acupuncture in improving the symptoms of PD patients. These studies have demonstrated the capacity of acupuncture to normalize brain functional connectivity, diminish neuronal apoptosis in the striatum, reduce lipid peroxide levels in dopaminergic neurons, and protect neurons from oxidative damage (7–9).

Nevertheless, the effectiveness of acupuncture as an independent treatment for individuals with PD remains uncertain. Given the ethical considerations favoring the use of drug therapies in PD, current clinical investigations have integrated acupuncture with medication-based treatments. At present, there is a lack of clinical observation reports specifically exploring the use of acupuncture in isolated treatment for PD. This case report presented a patient who declined anti-Parkinson drugs and opted solely for acupuncture treatment.

## 2 Case report

A 75-year-old male belonging to Huanggang, Hubei province, previously engaged in farming with a history of pesticide and herbicide exposure. He had no prior history of hypertension, diabetes, or cerebrovascular incidents, and there was no family history of PD. In March 2022, he developed unprovoked left-hand tremors, prompting his family to seek medical attention at Wuhan Union Hospital of China, where he received a diagnosis of PD. The patient declined anti-Parkinson drugs due to their lifelong requirement and common side effects, and the disease progressed, resulting in right-hand tremors as well.

On 12 June 2023, he visited the outpatient clinic of our hospital, seeking acupuncture therapy. At this time, he displayed characteristic PD symptoms, including limb tremors, rigidity, bradykinesia, and fatigue (Supplementary Video S1), which confirmed the PD diagnosis. Beyond motor issues, he experienced considerable stiffness from the neck to the back, difficulty in swallowing hard food, drooling on head lowering, occasional nocturnal foot cramps, dry mouth, and constipation. The patient had not started any medication, and no surgical treatment for deep brain stimulation had been performed prior to the acupuncture treatment.

**Figure 1:**
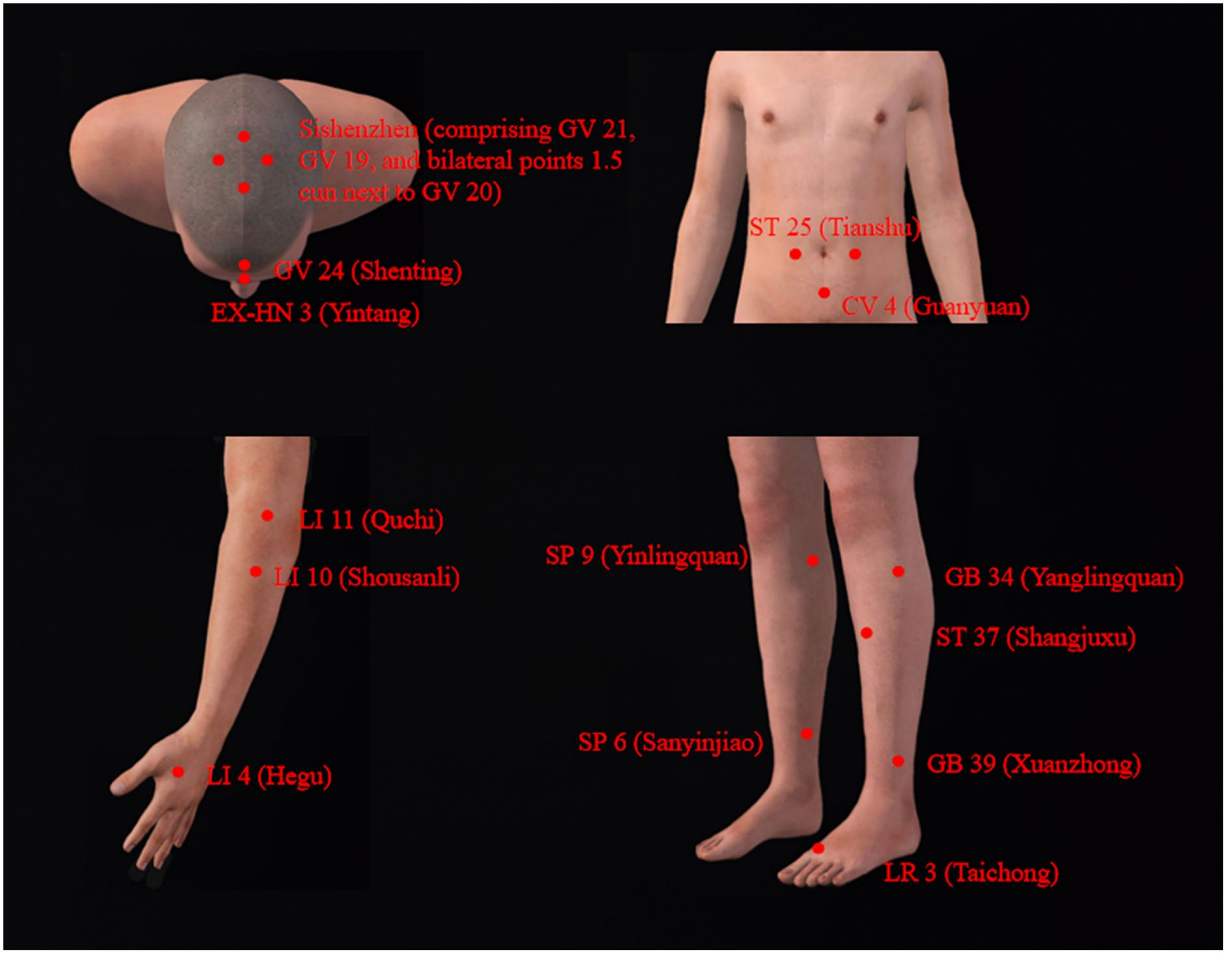
Acupuncture points selected in the supine position.

This patient displayed evident motor and non-motor symptoms. We employed two distinct combinations of acupoints for alternating treatment positions. In the supine position, as shown in Figure 1, acupuncture therapy primarily targets specific acupoints within the Shen-regulation acupuncture point set, with the selection of other acupoints tailored in accordance with presenting symptoms. The Shen-regulation acupuncture point set predominantly encompasses several key acupoints, namely, Sishenzhen (comprising GV 21, GV 19, and bilateral points 1.5 cun next to GV 20), GV 24 (Shenting), EX-HN 3 (Yintang), SP 6 (Sanyinjiao), LI 4 (Hegu), and LR 3 (Taichong). This specific acupuncture point set has been employed in previous clinical studies, demonstrating its effectiveness in alleviating clinical symptoms among individuals diagnosed with PD (10, 11). Supplementary acupoints such as ST 25 (Tianshu), CV 4 (Guanyuan), and ST 37 (Shangjuxu) are included based on the presentation of constipation symptoms (10). For limb tremors, acupoints such as LI 11 (Quchi), LI 10 (Shousanli), GB 34 (Yanglingquan), SP 9 (Yinlingquan), and GB 39 (Xuanzhong) have been integrated.

**Figure 2:**
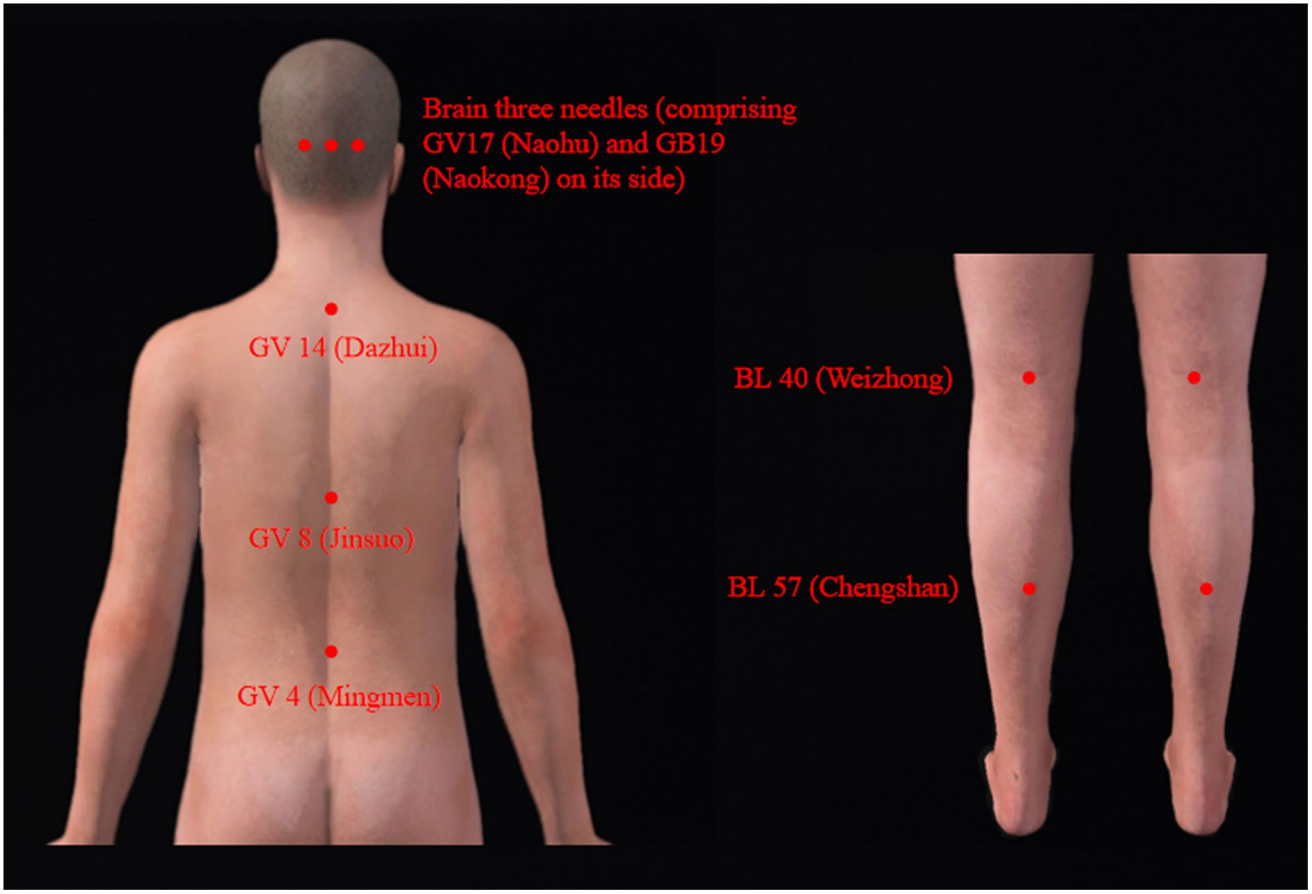
Acupuncture points selected in the prone position.

Acupuncture administered in the prone position was primarily focused on regulating the balance function of the patient. The selection of acupuncture points (Figure 2) was primarily guided by previous clinical expertise and experience (12–14). These include the utilization of the brain three needles, comprising three acupoints: GV 17 (Naohu) and GB 19 (Naokong) on its side, as well as Du three needles [three acupoints, consisting of GV 14 (Dazhui), GV 8 (Jinsuo), and GV 4 (Mingmen)]. Additional acupoints integrated into the treatment protocol were BL40 (Weizhong) and BL57 (Chengshan).

Acupuncture was performed with disposable, sanitized stainless steel needles (Tianxie, Suzhou Medical Appliance Factory, Suzhou, China; 0.25 × 25 mm, 0.25 × 40 mm). After routine disinfection, the acupuncturist inserted the needles into the corresponding acupoints. Head acupoints were treated with transverse insertion, back acupoints were treated with oblique insertion, and other points were treated with perpendicular insertion. The needles were twisted to stimulate qi flow, and the patient obtained the de qi sensation (a combination of feelings, such as numbness, soreness, and heaviness). All needles were kept in place for 30 min post-qi arrival. The acupuncture treatment was administered three times a week for 1 month, totaling 12 sessions.

Before and after acupuncture treatment, we appraised the condition of the patient across the three dimensions. Initially, concerning PD severity and health-related quality of life, post-acupuncture treatment revealed a 17-point reduction in UPDRS score, including an 11-point decrease in Part II. The PDQ-39 score exhibited an 11-point reduction.

Subsequently, non-motor symptoms were evaluated using the Non-Motor Symptoms Scale (NMSS) and the Hamilton Anxiety Scale (HAMA). The patient predominantly exhibited evident fatigue, diminished mood, and gastrointestinal issues (swallowing difficulty, drooling, and constipation). Comparative assessment indicated a 4-point enhancement in fatigue, a 36-point improvement in mood, and the complete resolution of gastrointestinal complications. The HAMA scores decreased by 5 points.

Third, motor function, primarily focusing on balance, was assessed using the Berg Balance Scale, demonstrating a seven-point increase. Further measurements of static and dynamic balance were conducted using Footscan V9 and TUGT, respectively. The 30-m walking test revealed a 0.11 m/s increase in walking speed and a 6 cm increase in stride length. Detailed outcomes and their associated values for minimal clinically important differences (MCID) are presented in Table 1.

**Table 1:** The parameter changes before and after acupuncture treatment.

Substance			Before treatment	After 1 month	D	MCID
NMSS		Cardiovascular	0	0	0	–
		Sleep/fatigue	8	4	−4	–
		Mood/cognition	37	1	−36	–
		Perceptual problems/hallucinations	0	0	0	–
		Attention/memory	0	0	0	–
		Gastrointestinal tract	20	0	−20	–
		Urinary	0	0	0	–
		sexual function	0	0	0	–
		miscellaneous	0	0	0	–
		Total	65	5	−60	–
HAMA			10	5	−5	−4 (11)
UPDRS		I	4	3	−1	–
		II	20	9	−11	–
		III	39	34	−5	−5 (15)
		Total	63	46	−17	−8 (15)
PDQ-39			19	8	−11	−4.72 (16)
Berg			42	49	7	6.5 (17)
Static balance	Eyes open	Enveloped area (mm)	7	2	−5	–
		Xd	7	5	−2	–
		Yd	12	6	−6	–
	Eyes closed	Enveloped area (mm)	7	5	−2	–
		Xd	5	5	0	–
		Yd	13	8	−5	–
TUGT			16.40	13.75	−2.65	–
The 30-m walk test		Speed (m/s)	0.70	0.81	0.11	–
		stride length (m)	0.42	0.48	0.06	–

MCID, minimal clinically important difference; D, the difference between 1 month after treatment and before treatment; UPDRS, Unified Parkinson’s Disease Rating Scale; PDQ-39, 39-item Parkinson Disease Questionnaire; Berg, Berg Balance Scale; TUGT, the Timed Up and Go test; NMSS, the Non-Motor Symptoms Scale; HAMA, Hamilton Anxiety Scale.

After 1 month of the treatment, the patient reported significant improvements in mood, reduced drooling, notably decreased stiffness in the neck and back, and overall alleviation of other symptoms. Additionally, he expressed enhanced confidence in managing PD. Subsequent telephone follow-up findings after the 1-month acupuncture treatment indicated an absence of recurring non-motor symptoms such as choking, drooling, and cramps.

## 3 Discussion

### 3.1 Non-pharmacological approaches in PD management

The management of PD predominantly revolves around pharmaceutical interventions (3, 4). However, the constraints associated with drug-based therapies (2) have prompted clinicians to explore the amalgamation of non-pharmacological modalities such as acupuncture (18, 19), cognitive behavioral therapy (20), physical exercise (21), and music therapy (22) alongside pharmacotherapy. This combined approach aims to attenuate adverse effects while enhancing therapeutic outcomes in the care of individuals afflicted by PD.

Exercises that incorporate goal-based training and aerobic activity hold promise for augmenting cognitive and automatic components of motor control in individuals with mild to moderate PD through experience-dependent neuroplasticity (21). Clinical studies have evidenced the capacity of physical exercise to modify brain function and structure among PD patients, leading to the stabilization of disease progression within the corticostriatal sensorimotor network and contributing significantly to bolstering cognitive abilities (23). Sleep dysfunction observed in PD can also show signs of improvement through the intervention of exercise (24).

Cognitive behavioral therapy (CBT), a comprehensive therapeutic approach employing diverse techniques and concepts (21), stands as a valuable intervention aiding individuals in navigating the multifaceted challenges linked to Parkinson’s disease (PD), thereby fostering heightened well-being and an augmented overall quality of life. Evidence suggests its efficacy in ameliorating depressive and anxious symptoms, which are prevalent in individuals with PD (23, 24). A study integrating CBT among PD patients showed promise in alleviating neuropsychiatric disturbances and reducing symptom severity (25).

Acupuncture therapy, supported by a number of clinical studies, demonstrates efficacy in ameliorating motor symptoms such as myotonia in PD patients (12). Additionally, it addresses prevalent non-motor symptoms including anxiety (11), constipation (10), and insomnia (26). Notably, the therapeutic impact surpasses placebo acupuncture, highlighting its significant clinical efficacy (10, 11). Several recently published meta-analyses also suggested that acupuncture-related therapies combined with conventional medication showed a moderate or large effect on movement function in patients with PD (6, 27). Animal studies have established a scientific foundation supporting the potential of acupuncture in ameliorating symptoms observed in Parkinson’s disease (7–9).

In a comprehensive meta-analysis evaluating non-pharmacological interventions for adult depression, significant superiority over placebo was observed for physical exercise, CBT, and acupuncture. Additionally, the analysis indicated that acupuncture exhibits superior effectiveness in managing major depressive disorder when compared with exercise therapy and CBT (28).

### 3.2 Efficacy of acupuncture as a standalone therapy for PD improvement

Ethically, drug utilization is standard in PD care, and consequently, ongoing studies have integrated acupuncture as an adjunct to medication-based treatments. Given the substantial impact of anti-Parkinson drugs on PD patients, the efficacy of acupuncture as a standalone treatment for PD remains uncertain.

The therapeutic effects of acupuncture treatment solely on the PD symptoms of this patient encompass three key aspects. First, significant enhancements in UPDRS and PDQ-39 scores exceeded their respective minimal clinically important difference (MCID) values, demonstrating the clinical relevance of acupuncture as an independent approach for ameliorating PD symptoms. Second, the non-motor symptoms of the patient, predominantly involving fatigue, mood, and gastrointestinal challenges, notably improved. Fatigue decreased, mood enhanced significantly with greater initiative, and symptoms of drooling and choking on hard food disappeared. The reduction in the HAMA score surpassed the MCID, indicating the clinical importance of acupuncture in alleviating anxiety. This is consistent with previous findings (11, 29). Third, improvements in motor symptoms included enhanced static and dynamic balance, supported by the Berg scale, TUGT test, and Footscan V9 findings. The 30-m walking test showed improved stride length and step count. Visual enhancements in autonomous arm movement and left-hand vibration amplitude were observed in the video analysis. Subsequent follow-up demonstrated the safety and effectiveness of acupuncture treatment, with no post-improvement symptom relapse.

The particular case in this report highlights that acupuncture alone could effectively improve the motor and non-motor symptoms in this patient. This underscores the potential of acupuncture as an alternative or complementary therapy for individuals who refuse or are not suitable for conventional anti-Parkinson drugs, providing an additional option for symptom management. The absence of recurring non-motor symptoms post-acupuncture suggests potential long-term benefits. However, further studies are needed to assess its long-term efficacy.

### 3.3 Medication refusal in PD: factors and mitigation

In clinical settings, few patients decline treatments that could potentially enhance their wellbeing and quality of life (30). Instances of medication refusal among individuals with neurodegenerative conditions are infrequent but do occur (31).

The causes behind medication refusal in PD are varied. Treatment for PD often involves lifelong medication, commonly leading to side effects such as impulse control disorders (32) and Levodopa-induced dyskinesia (33). Patients frequently require adjustments in levodopa dosage as the disease advances (34), while healthcare expenses also negatively affect medication adherence (35). These challenges may directly lead to patient refusal and indirectly contribute to heightened anxiety and stress.

Enhancing physician–patient communication could reduce the incidence of treatment refusal (36). Psychological interventions also proved beneficial in enhancing the adherence of patients to medication regimens (37).

Animal experiments found that electroacupuncture could act on depression by modulating the HPA axis and enhancing hippocampal 5-HT/5-HT1AR in chronic unpredictable mild stress rats (29). An additional clinical randomized controlled trial of PD patients with anxiety yielded the same results (11).

In this case, acupuncture as a standalone therapy demonstrated significant mood enhancement in the individual with PD, indicating its potential efficacy for PD patients who decline medication due to emotional issues.

Regrettably, as the hometown of the patient was in Hubei Province, he faced various inconveniences during the treatment period while residing near the hospital, impacting his daily life and limiting the possibility of continued long-term treatment. With only 12 acupuncture sessions received, further studies are necessary to comprehensively evaluate the long-term effectiveness of acupuncture treatment for PD.

## Data availability statement

The raw data supporting the conclusions of this article will be made available by the authors, without undue reservation.

## Ethics statement

The study has been reviewed and approved by the Ethics Committee of the First Affiliated Hospital of Guangzhou University of Chinese Medicine (Ethics number: K-2023-127). Written informed consent from the patient was not required to participate in this study in accordance with the national legislation and the institutional requirements. Written informed consent was obtained from the individual for the publication of any potentially identifiable images or data included in this article.

## Author contributions

SL: Methodology, Writing – original draft, Writing – review & editing. QL: Investigation, Writing – original draft. IL: Supervision, Writing – original draft. JF: Validation, Writing – review & editing. YT: Resources, Writing – original draft. XL: Data curation, Writing – review & editing. XX: Investigation, Writing – original draft. LZ: Funding acquisition, Resources, Writing – review & editing.
